# mRNA-based seasonal influenza vaccines: an overview of immunogenicity, efficacy, and safety

**DOI:** 10.3389/fimmu.2026.1965512

**Published:** 2026-09-14

**Authors:** Alexander Domnich, Andrea Orsi

**Affiliations:** 1 AOM – IRCCS Ospedale Policlinico San Martino, Genoa, Italy; 2 Department of Health Sciences (DISSAL), University of Genoa, Genoa, Italy; 3 Interuniversity Research Centre on Influenza and Other Transmissible Infections (CIRI-IT), Genoa, Italy

**Keywords:** influenza, influenza vaccines, modRNA, mRNA vaccines, mRNA-1010, mRNA-1083

## Abstract

Available seasonal influenza vaccines, most of which are still produced in eggs, have several limitations. Messenger RNA (mRNA) technology has emerged as a transformative approach capable of overcoming some of these shortcomings. This overview synthesizes clinical trials evaluating the immunogenicity, efficacy, and safety of standalone and combination mRNA influenza vaccines. Moderna’s mRNA-1010 (now approved in the United States) and Pfizer’s modified-nucleoside RNA candidate (modRNA) represent the most advanced standalone platforms, while Moderna’s combination influenza and COVID-19 vaccine, mRNA-1083, was recently granted European authorization. Following iterative platform optimization, these vaccines induce robust humoral and cell-mediated responses, especially against influenza A strains, characterized by extended germinal center reactions, continuous somatic hypermutation, enhanced Fc-mediated effector functions, and strong T helper 1 engagement. In phase III relative efficacy trials, an optimized mRNA-1010 formulation achieved superiority over standard-dose vaccines with a relative efficacy of 26.6% (95% CI: 16.7%, 35.4%) in adults aged ≥50 years. Pfizer’s first-generation quadrivalent modRNA candidate showed 34.5% (95% CI: 7.4%, 53.9%) relative efficacy against a standard-dose comparator in younger adults aged 18–64 years, but failed to demonstrate non-inferiority in older adults (≥65 years), showing a relative efficacy of −5.8% (95% CI: −47.2%, 23.8%). However, whether mRNA platforms offer incremental benefits over enhanced formulations (such as high-dose, adjuvanted, or recombinant vaccines), which are the preferred options in some countries for older adults, remains to be established. The safety profile aligns with licensed COVID-19 mRNA vaccines; although reactogenicity is higher than with conventional vaccines, adverse reactions are predominantly mild-to-moderate and transient. Operationally, transitioning to single-dose pre-filled syringes alongside expanding refrigerated stability data significantly mitigates historical cold-chain bottlenecks. However, while standalone and combination mRNA platforms represent a major technological advance, real-world implementation challenges may affect their broader global health impact.

## 1 Introduction

Seasonal influenza remains a leading infectious cause of global morbidity and mortality, characterized by high attack rates across all age groups (1). Accurately quantifying the true global burden of influenza is challenging due to disparities in the availability of high-quality epidemiological data and wide variations in national surveillance systems. Nevertheless, the Global Burden of Disease study estimated that in 2017 alone, influenza was responsible for approximately 9.5 million hospitalizations and 145,000 deaths due to lower respiratory tract infections (2). Modeling by Iuliano et al. suggests an even higher global impact, estimating annual influenza-associated respiratory mortality between 291,243 and 645,832 deaths (3). Furthermore, the burden of influenza extends well beyond respiratory illness; through multiple systemic inflammatory and mechanistic pathways, influenza virus increases the risk of severe extra-respiratory complications, including major adverse cardiovascular events, acute neurological syndromes, renal failure, and other organ dysfunctions (4).

Annual vaccination remains the cornerstone of public health strategies to reduce the global burden of influenza. Accordingly, the World Health Organization (WHO) position paper on seasonal influenza vaccines (SIVs) underscores that vaccination efforts should prioritize individuals at high risk of severe complications (e.g., older adults, pregnant women, young children, and individuals with underlying chronic comorbidities) as well as those with high exposure or transmission potential, such as healthcare professionals (5). However, the protection conferred by currently available SIVs remains suboptimal across all major age-classes, including children (6), working-age adults (7), and older populations (8). For example, in the post-COVID-19 pandemic period, real-world SIV effectiveness in the United States (US) ranged from 30% in the 2022/2023 season to 56% in the 2024/2025 season (9). Vaccine effectiveness is driven by a complex interplay of viral (e.g., rapid antigenic drift, vaccine-strain mismatch, altered glycosylation patterns), host (e.g., immunosenescence, imprinting, complex effects of prior vaccination), and SIV-related (e.g., emergence of egg-adaptive mutations, variable antigen content) factors (10, 11). Nevertheless, annual vaccination of high-risk populations remains a cost-effective intervention, even during seasons marked by low vaccine effectiveness (12).

The WHO Global Influenza Strategy 2019–2030 underscores a public health imperative to develop next-generation SIVs that offer stronger, broader, and more durable protection, alongside accelerated manufacturing timelines (13). In defining the preferred product characteristics for improved SIVs, the WHO highlights that novel vaccines should demonstrate superior efficacy, with a minimum duration of protection of one year (with a preference for ≥3 years) and an expanded breadth of coverage against all circulating viral (sub)types. Furthermore, to effectively mitigate the threats of antigenic drift or pandemic emergence, next-generation platforms should be manufactured in less than five months from strain selection to the finished product (14). In this context, the messenger ribonucleic acid (mRNA) platform emerges as a promising candidate capable of addressing some of these benchmarks.

As demonstrated by the COVID-19 pandemic, mRNA technology and lipid nanoparticle (LNP) delivery represent a significant advance in vaccinology, integrating accelerated development, scalable manufacturing, robust immunogenicity, and a favorable safety profile. Notably, influenza viruses were targeted by mRNA vaccine technology prior to the COVID-19 pandemic; however, investigation was primarily restricted to preclinical or early stages pending necessary optimizations in mRNA stability and formulation delivery. Following the success of SARS-CoV-2 mRNA vaccines, the regulatory approval of the respiratory syncytial virus (RSV) mRNA vaccine (mRNA-1345) marked an important expansion of the platform, paving the way for broader applications across other infectious disease targets (15). While not intended to be exhaustive, this overview outlines the evolving application of mRNA technology platforms to seasonal influenza prevention. Particular emphasis is placed on characterizing the immunogenicity, efficacy, and safety observed during the clinical development of these vaccines.

## 2 Currently available non-mRNA seasonal influenza vaccines and their limitations

Currently available SIVs are trivalent formulations designed to protect against circulating influenza A(H1N1)pdm09, A(H3N2), and B/Victoria viruses (16). Quadrivalent SIVs, which were introduced in 2013 to additionally cover the B/Yamagata lineage (17), are no longer recommended or manufactured due to the apparent global extinction of this B lineage (18, 19). Although vaccine availability and regulatory approvals vary by country, three primary categories of SIVs are currently available: egg- or cell-culture-based inactivated vaccines, live attenuated vaccines, and recombinant vaccines (20).

### 2.1 Standard egg-based vaccines

Historically, whole-virion inactivated vaccines were the first to enter large clinical use. Following viral propagation in embryonated chicken eggs, virions are inactivated using chemical or physical methods. To improve reactogenicity profiles, split-virion and subunit vaccines progressively replaced whole-virion formulations starting in the 1960s (21, 22), although whole-virion vaccines remain in limited regional use (23). Split-virion vaccines are manufactured from whole-virion precursors by applying detergents to disrupt the viral membrane, followed by detergent removal via tangential flow filtration. Subunit vaccines undergo additional purification to isolate the major surface glycoproteins, hemagglutinin (HA) and neuraminidase (NA), while removing internal viral proteins (24, 25). A meta-analysis of randomized controlled trials (RCTs) demonstrated comparable hemagglutination-inhibition (HAI) antibody titers between subunit and split-virion vaccines, with lower frequencies of local and systemic adverse reactions (ARs) observed in subjects receiving subunit formulations (26). Conversely, split-virion vaccines have been reported to elicit broader cell-mediated immune responses (27) and demonstrate a slightly higher field effectiveness (28). These differences may be attributed to the presence of internal influenza proteins within split-virion formulations, which serve as primary targets for cell-mediated immunity (29). Intramuscularly administered split-virion and subunit vaccines containing 15 *µ*g of HA per strain are typically indicated as standard-dose inactivated SIVs (sdIIVs). Conversely, NA content is not standardized, varying significantly by viral strain and manufacturer (30). Most available sdIIVs are indicated for individuals aged ≥6 months (16).

### 2.2 Enhanced vaccines

As age advances, both the innate and adaptive immune systems undergo progressive involution, a process termed immunosenescence, resulting in a diminished capacity to respond to novel antigens and a decline in long-term immunological memory. Consequently, older adults mount weaker and less durable humoral and cellular responses following vaccination (31). Indeed, a quantitative review demonstrated that, compared to individuals aged 18–59 years, those aged ≥60 years have a 70%–80% lower likelihood of achieving seroconversion (defined as a ≥4-fold post-vaccination increase in HAI titers) against any influenza strain included in the vaccine (32). To overcome the reduced immunogenicity of sdIIVs in older populations, two main strategies have been developed: (i) adding adjuvants while maintaining a standard antigen dose of 15 *µ*g per strain, and (ii) increasing the amount of antigen to 45 *µ*g or 60 *µ*g per strain. Implementing these approaches has led to the development of three “enhanced” SIV platforms: adjuvanted standard-dose egg-based inactivated vaccine (aIIV), high-dose egg-based inactivated vaccine (hdIIV), and high-dose recombinant influenza vaccine (RIV) (33). As an egg-free formulation, RIV will be discussed later in the text. Previously, a split intradermal vaccine was also leveraged to enhance responses, as the dermis is particularly rich in dendritic cells capable of efficiently generating innate and adaptive immune responses, though it has since been discontinued (34). In several jurisdictions, including the US (16), Canada (35), the United Kingdom (UK) (36), and some European countries (33), enhanced SIVs are preferentially recommended over sdIIVs for older adults.

### 2.3 MF59-adjuvanted egg-based vaccine

The aIIV platform is a subunit sdIIV formulation containing the MF59 adjuvant. MF59 is an opalescent oil-in-water emulsion composed of squalene microfluidized within a citrate buffer to create uniform spherical surfactant-stabilized droplets. The mechanism of action of MF59 is multifaceted: at the injection site, it enhances the recruitment of monocytes and macrophages, thereby optimizing local antigen uptake and processing. These recruited immune cells secrete chemokines and cytokines that amplify downstream signaling. MF59 promotes and accelerates the differentiation of antigen-presenting cells (APCs), facilitating more efficient antigen transport to draining lymph nodes, where a broader array of B and T lymphocytes with more differentiated phenotypes are subsequently activated (37, 38). Indeed, the pooled post-vaccination HAI titers for the trivalent aIIV (aIIV3) versus trivalent sdIIV (sdIIV3) are 15%–30% higher across all homologous vaccine-like strains (39). Furthermore, compared to non-adjuvanted formulations, aIIV3 elicits a broader repertoire of antibody clones targeting the HA subunit 1 (HA1), which harbors the majority of neutralizing epitopes. Consequently, aIIV3 may provide broader cross-protective immunity, demonstrating enhanced immunogenicity against antigenically drifted strains not included in the seasonal formulation (40, 41). Improved immunogenicity against heterologous strains has been extensively demonstrated for the influenza A(H3N2) subtype, which is particularly prone to antigenic drift (39, 42, 43). In a RCT, aIIV3 has also been shown to induce higher anti-NA antibody responses compared to both quadrivalent sdIIV (sdIIV4) and trivalent hdIIV (hdIIV3) (44). In a recent network meta-analysis, the pooled relative effectiveness of aIIV versus sdIIV in preventing influenza-related hospitalizations was 20.0% [95% confidence interval (CI): –54.2%, 58.5%] in randomized studies and 10.3% (95% CI: 5.5%, 14.7%) in observational studies (45). However, no individually randomized studies assessing the relative vaccine efficacy (rVE) of aIIV against sdIIV or any other vaccine have been published to date. Regarding tolerability, aIIV3 is more reactogenic than sdIIV3, particularly at the injection site, with a 71% and 33% increase in the risk of solicited local and systemic ARs, respectively (46). Conversely, the relative risk (RR) of serious adverse events (SAEs) is comparable to that of sdIIVs (RR 0.95; 95% CI: 0.19, 4.72) (47). Currently, the age indication for aIIV3 is ≥50 years in Europe (48) and ≥65 years in the US (16).

### 2.4 High-dose egg-based vaccine

The alternative strategy for overcoming immunosenescence is increasing the antigenic payload. However, the clinical development of high-dose formulations requires a delicate balance: the gain in immunogenicity must be achieved while maintaining an acceptable reactogenicity, tolerability, and safety profile, thereby preserving a favorable risk-benefit ratio. The hdIIV platform is a non-adjuvanted, split-virion vaccine containing four times the standard antigen quantity (60 *µ*g of HA per strain compared to 15 *µ*g) (49). In terms of HAI responses toward vaccine-like strains, hdIIV3 is 74%, 84%, and 47% more immunogenic than sdIIV3 for A(H1N1), A(H3N2), and B, respectively (50). The relative increase for both influenza A strains with hdIIV3 versus sdIIV is higher than the corresponding increase observed in comparisons between aIIV3 and sdIIV (51). There is also some evidence supporting improved heterologous immunogenicity over sdIIV (42), although this evidence is more limited and less consistent (52) than that reported for aIIV3. In the pivotal RCT with individual allocation, hdIIV3 demonstrated a rVE of 24.2% (95% CI: 9.7%, 36.5%) compared to sdIIV3 against laboratory-confirmed influenza (53). In the aforementioned network meta-analysis, the relative effectiveness of hdIIV versus sdIIV was estimated at 25.1% (95% CI: –18.6%, 52.7%) in randomized studies and 10.2% (95% CI: 7.8%, 12.6%) in observational studies (45). A large individual-level pooled analysis showed a quadrivalent hdIIV (hdIIV4) significantly reduced hospitalization for influenza/pneumonia (by 8.8%), cardiorespiratory causes (by 6.3%), laboratory-confirmed influenza (by 31.9%), and any cause (by 2.2%), compared to sdIIV4, though all-cause mortality rates remained similar (54). Head-to-head evidence comparing hdIIV and aIIV remains more limited and inconclusive (55). For instance, pooled network estimates from Ferdinands et al. (45) indicated no significant difference in effectiveness across both randomized (hdIIV vs. aIIV: 6.3%; 95% CI: –108.7%, 58.0%) and non-randomized (aIIV vs. hdIIV: 0.0%; 95% CI: –5.3%, 5.0%) studies. From a reactogenicity standpoint, hdIIV is associated with significantly higher rates of local ARs (RR 1.40) and some systemic symptoms, including headache (RR 1.35), chills (RR 1.73), and malaise (RR 1.28) (56), while showing rates of SAEs comparable to sdIIVs (RR 1.02; 95% CI: 0.42, 2.46) (47). The age indication for hdIIV3 is lower in Europe [currently ≥60 years (57) with an anticipated expansion to ≥50 years (58)] than in the US (≥65 years) (16).

### 2.5 Live attenuated vaccine

Apart from the live attenuated influenza vaccine (LAIV) historically used in Russia, the first and only LAIV developed in Western countries was authorized in the US in 2003, utilizing a manufacturing platform distinct from those of inactivated vaccines (59). Each strain included in the vaccine is a 6:2 genetic reassortant, containing six genes encoding internal proteins derived from master donor viruses and two genes encoding HA and NA. Specifically, the development of LAIV was enabled by the discovery of cold-adaptation in type A and type B strains. Adaptation is achieved through serial passage in primary avian cell culture under progressively reduced thermal regimes. During this process, the viruses acquire key mutations in the genes encoding non-glycosylated internal proteins. Consequently, master donor strains are generated that possess cold-adapted, temperature-sensitive, and attenuated phenotypes. Subsequently, vaccine formulations are updated according to WHO recommendations using reverse genetics, inserting the HA and NA sequences from circulating wild-type strains into the master donor viruses (60–62). The mechanism of action of LAIV mimics natural infection, distinguishing it from inactivated platforms (60). Indeed, while protection induced by inactivated vaccines depends primarily on serum antibodies targeting HA and, to a lesser extent, NA (63), intranasally administered LAIV replicates predominantly in the ciliated epithelial cells of the nasopharyngeal mucosa. This induces robust mucosal immunoglobulin A (IgA) secretion along with enhanced CD4^+^ and CD8^+^ cellular responses; conversely, HAI titers are typically lower than those elicited by inactivated vaccines (60, 64). In RCTs, children vaccinated with trivalent LAIV (LAIV3) experienced 41%–50% fewer cases of influenza compared to those immunized with sdIIV3 (65). However, a recent meta-analysis of observational studies demonstrated that, compared to non-vaccinated pediatric populations, LAIV and sdIIV showed similar absolute effectiveness against any influenza (approximately 50%). Notably, across distinct seasons, LAIV demonstrated improved relative effectiveness against influenza B compared to sdIIV (relative effectiveness between 2017/2018 and 2022/2023: 196%; 95% CI: 73%, 406%), but reduced effectiveness against A(H1N1)pdm09 (relative effectiveness between 2010/2011 and 2016/2017: −46%; 95% CI: −57%, −33%) (66). A root-cause analysis performed to elucidate the reduced LAIV effectiveness against A(H1N1)pdm09 observed during certain seasons revealed that impaired replicative fitness of the vaccine strain increased its susceptibility to inter-strain competition within multivalent formulations, ultimately suppressing protection. To resolve this, candidate strain selection was updated to mandate multi-cycle replication testing in human nasal epithelial cells and ferret models before formulation (67). Overall, LAIV has favorable safety and tolerability profiles in both pediatric and young adult populations (59, 62), though its clinical application remains predominantly focused on pediatric immunization. Regulatory authorizations vary geographically; while indicated for individuals aged 2–49 years in the US (16), its approval in Europe is restricted to children and adolescents aged 2–17 years (68). Reflecting these indications, some health authorities, such as in the UK, preferentially recommend LAIV for eligible pediatric cohorts aged 2–17 years (36).

### 2.6 Limitations of the egg-based vaccines

Worldwide, about 90% of the entire SIV production capacity is represented by egg-based vaccines (20) and all SIV types discussed so far were egg-derived. Although the traditional egg-based platform is time-proven, highly standardized, and inexpensive, it is also associated with several limitations. First, it inherently relies on the timely supply of large quantities of eggs that are prequalified for the biotechnology industry. Certain events, such as a widespread avian influenza outbreak in poultry, can compromise the availability of eggs as a raw material. Second, process scalability is suboptimal and the volume of SIV produced is planned far in advance, which does not allow for a drastic increase in response to a sudden surge in demand. Third, although embryonated eggs naturally filter out many viruses to lower the risk of adventitious agents, the manual inoculation and harvesting process remains prone to contamination, requiring the frequent use of preservatives and antibiotics (69, 70). The fourth drawback concerns a potential emergence of egg-adaptive mutations during vaccine production. Candidate vaccine viruses (CVVs) selected by the WHO are obtained either by direct egg passage or through reassortment with high-growth donor strains in eggs to generate viable production seeds (71). During egg propagation, the abundance of avian-type (*α*-2,3Gal) receptors often results in the selection of variants that increase binding to avian-type receptors on the chorioallantoic membrane while reducing binding to human-type (*α*-2,6Gal) receptors. These egg-adaptive mutations can alter antigenicity and therefore reduce SIV effectiveness, particularly for the A(H3N2) subtype (72). For example, circulating A(H3N2) strains recently acquired a novel *N*-glycosylation sequon within HA antigenic site B, which compromised viral growth in eggs. This created strong selective pressure during manufacturing for egg-adapted variants that lost the glycan to optimize host cell attachment. Consequently, these non-glycosylated egg-adapted vaccine strains exposed immunodominant epitopes that elicited antibodies with reduced capacity to recognize or neutralize the glycosylated wild-type viruses circulating in the human population (73). Therefore, the use of alternative manufacturing platforms circumvents these structural bottlenecks by eliminating egg-adaptive mutations.

### 2.7 Cell-based standard-dose vaccine

A trivalent cell-based standard-dose inactivated subunit SIV (cIIV3) was first authorized in Europe in 2007. However, in line with WHO recommendations at that time, the vaccine was still manufactured using egg-derived CVVs (74). Beginning in the 2019/2020 season, all vaccine strains have been isolated and propagated exclusively in cell culture, rendering the formulation completely egg-free. The cell-based platform is manufactured using a proprietary Madin-Darby Canine Kidney (MDCK) mammalian cell line (71), which was genetically modified to increase the surface density of *α*-2,6Gal receptors (75). Viral propagation occurs within closed-system bioreactors and, unlike traditional egg-based processes, does not require antibiotics due to a negligible risk of bacterial contamination. Subsequent ultracentrifugation, filtration, and chromatography steps effectively eliminate MDCK cell components from the final purified product (76). Although cell-adaptive mutations can occur, they are infrequent and randomly scattered across the HA protein, reflecting lack of proofreading rather than MDCK-driven adaptation (77). Compared to egg-based sdIIV3, cIIV3 demonstrates non-inferior post-vaccination antibody titers and shows a comparable reactogenicity profile across all age groups (74, 78). In RCTs evaluating laboratory-confirmed influenza endpoints, the absolute efficacy of cIIV3 was 69.5% [one-sided 97.5% CI lower limit (LL): 55.0%] in adults aged 18–49 years (79), whereas the absolute efficacy of its quadrivalent counterpart (cIIV4) in young children (6–47 months) was 41.3% (97.98% CI: 21.6%, 56.0%) (80). RCTs evaluating rVE are currently unavailable (78). Although alignment data suggest that cell-propagated influenza A(H3N2) and B/Victoria reference viruses retain greater antigenic similarity to circulating wild-type strains compared to egg-propagated reference viruses (81), real-world relative effectiveness data remain inconclusive (78) and appear to vary by season and age group (82). For instance, one systematic review (82) indicated that cIIV4 was significantly more effective than egg-based sdIIVs during the 2017/2018 season (relative effectiveness 11%; 95% CI: 8%, 14%), but not during the 2018/2019 season (relative effectiveness 3%; 95% CI: –2%, 7%). Similarly, a small but significant advantage was observed in younger adults (7%; 95% CI: 4%, 10%), but not in the elderly (3%; 95% CI: –4%, 10%). cIIV3 holds a universal age indication (≥6 months) (16).

### 2.8 MF59-adjuvanted high-dose cell-based vaccine

In July 2026, a trivalent high-dose cell-based subunit inactivated vaccine adjuvanted with MF59 (acIIV3) was authorized in Europe (83) and the UK (84) for adults aged ≥50 years. Compared to aIIV3, acIIV3 features a 3-fold increase in HA antigen payload (45 *µ*g per strain) and a 2-fold higher content of the MF59 adjuvant (84). During early clinical development, its quadrivalent formulation (acIIV4) demonstrated superior immunogenicity relative to cIIV4 across all evaluated strains (85–87). In the pivotal phase III RCT, adults aged ≥50 years immunized with acIIV4 mounted stronger HAI responses compared to those receiving aIIV4, while demonstrating responses comparable to RIV4 against A(H1N1)pdm09, numerically higher titers for B/Victoria and B/Yamagata, but lower titers against A(H3N2) (84).

### 2.9 Recombinant vaccine

The second egg-free vaccine platform utilizes cell culture combined with the recombinant the Baculovirus Expression Vector System technology. Baculoviruses are insect pathogens whose DNA genome can be genetically engineered within specialized insect cell cultures, such as those derived from the fall armyworm moth *Spodoptera frugiperda* (SF) (88). Based on WHO recommendations for SIV composition, the HA gene sequence for each target strain is cloned and expressed within the recombinant baculovirus vector. The recombinant virus then infects the *expres*SF+ insect cell culture, which drives high-yield expression of the recombinant HA protein. The recombinant HA molecules are subsequently extracted and purified through a series of chromatography and filtration steps. The entire process ensures complete genetic fidelity, as the expressed recombinant HA sequence matches the WHO-selected reference strain perfectly, thereby eliminating egg-adaptive mutations. RIV formulation contains three times the standard HA antigen payload (45 *µ*g of HA per strain) but, unlike other vaccines, contains no NA and therefore relies exclusively on anti-HA immunity (88, 89). Trivalent RIV (RIV3) is primarily available in the US, where it is authorized for individuals aged ≥9 years and holds a preferential recommendation (alongside hdIIV and aIIV) for adults aged ≥65 years (16). RIV is highly immunogenic across diverse age-classes (90). However, higher post-vaccination HAI titers relative to sdIIV were predominantly observed for influenza A, particularly A(H3N2), and not for influenza B (91–94). Indeed, in older adults, the quadrivalent RIV formulation (RIV4) demonstrated a superior geometric mean fold rises (GMFRs) in HAI titers from baseline to one-month post-vaccination against influenza A(H3N2) compared not only to sdIIV4, but also to aIIV3 and hdIIV3 (93). In the pivotal RCT conducted among adults aged ≥50 years, RIV4 demonstrated superior rVE over sdIIV4, reducing RT-PCR-confirmed influenza illness by 30% (95% CI: 10%, 47%) (94). Real-world observational evidence aligns with these trial findings: in a network meta-analysis of observational studies, the pooled relative effectiveness of RIV versus sdIIV, aIIV, and hdIIV in preventing influenza-related hospitalizations in the elderly was estimated at 18.5% (95% CI: 7.9%, 27.9%), 9.2% (95% CI: –2.9%, 19.9%), and 9.2% (95% CI: –2.6%, 19.7%), respectively (45). With the exception of a slightly higher incidence of chills (RR 1.33), the overall safety and reactogenicity profile of RIV is comparable to that of sdIIVs (95).

## 3 mRNA platform for seasonal influenza vaccines and its potential advantages

Vaccines based on nucleic acids contain antigens encoded by either DNA or RNA and have shown promise in combining some advantages of live attenuated and subunit vaccines (96). mRNA serves as an intermediate between a protein-coding gene and its protein product. This molecule is therefore non-infectious and non-integrating with no potential risk of insertional mutagenesis (97).

Injectable mRNA vaccines fundamentally rely on two essential elements: the antigen-encoding mRNA construct and a protective carrier that prevents ribonuclease degradation and mediates cellular uptake, enabling intact cytosolic delivery for subsequent translation (98, 99). Although novel alternative delivery platforms are actively being developed, all approved and late-stage mRNA vaccines employ LNPs (100, 101). Comprising a four-component lipid formulation of ionizable lipids, neutral phospholipids, cholesterol, and PEGylated lipids, LNPs possess distinct physicochemical properties, including particle size, surface charge, and PEG chain length, that profoundly modulate cellular uptake, lymphatic drainage, and endosomal escape (101).

Several mRNA vaccine platforms have been engineered, including conventional non-replicating linear mRNA (with or without nucleoside modifications), self-amplifying mRNA (saRNA), and emerging circular mRNAs (circRNA) or trans-amplifying platforms (102). *In vitro* transcribed non-replicating linear mRNA mimics the single-stranded structure of endogenous mRNA and is composed of five distinct regions: 5′ cap, 5′ untranslated region (UTR), open reading frame (ORF), 3′ UTR, and polyadenylated (polyA) tail (103). Both the 5′ cap and polyA are essential for translation initiation and transcript stabilization during cellular processes. Although the 5′ and 3′ UTRs do not directly contribute to protein expression, these regulatory elements are necessary for the modulation of mRNA translation, half-life, and subcellular localization. Finally, the ORF represents the central coding sequence specifying the primary amino acid sequence of the target antigen (98, 103, 104).

Linear mRNA vaccines can be synthesized using either unmodified or nucleoside-modified formats (98). The use of modified mRNA nucleosides has been the key to the development of COVID-19 vaccines: the first two licensed and widely deployed COVID-19 mRNA vaccines by Pfizer/BioNTech and Moderna utilized this format (100, 105). Conversely, unmodified uridine-containing mRNA are sensed by a number of Toll-like receptors (TLRs) and other intracellular sensors, which leads to a strong innate immune response and insufficient protein expression (100, 102). In a RCT, an unmodified mRNA COVID-19 vaccine showed lower absolute efficacy compared to nucleoside-modified platforms, which was attributed to dose restriction to limit inherent reactogenicity, alongside reduced translational efficiency yielding lower antigen expression levels (106). Negative effects of this uridine-dependent immune sensing can be mitigated by replacing uridine with naturally occurring pseudouridine or its synthetic derivative *N*^1^-methylpseudouridine (100, 102). This nucleoside substitution enhances transcript stability by suppressing hydrolysis at modified residues while markedly increasing protein expression (98).

The foundational principle of mRNA vaccines relies on host cells taking up foreign mRNA, translating it into specific viral proteins, and presenting these antigens to the immune system. Following intramuscular administration, mRNA-LNPs undergo receptor-mediated endocytosis by local APCs and subsequently escape from the endosomal compartment into the cytosol, where ribosomes translate the synthetic transcripts into target antigenic proteins. These intracellularly produced antigens engage adaptive immunity via distinct processing pathways. Specifically, proteasomal degradation yields peptide fragments loaded onto major histocompatibility complex (MHC) class I molecules to stimulate cytotoxic CD8^+^ lymphocytes, which eliminate target cells through the release of cytolytic effectors. Concurrently, secreted or membrane-bound antigens undergo endosomal processing and presentation on MHC class II molecules to CD4^+^ cells, driving cytokine-mediated phagocyte activation and B-cell differentiation for neutralizing antibody synthesis (103).

All mRNA influenza vaccines subsequently detailed in this manuscript utilize a proprietary linear nucleoside-modified mRNA construct; however, as we mentioned earlier, alternative platforms are under investigation, among which saRNA represents the most prominent strategy. Indeed, two COVID-19 saRNA vaccines (ARCT-154 and HCG019) have been approved in some jurisdictions (107). Engineered from alphavirus genomes, saRNA constructs retain the non-structural genes encoding the RNA-dependent RNA polymerase while replacing the structural genes with the target antigen sequence. Upon cytosolic delivery, saRNA leverages this alphaviral replication machinery to generate abundant copies of both the full-length genomic transcript and the subgenomic mRNA encoding the antigen. This intrinsic self-amplification enables robust antigen expression at reduced doses relative to conventional non-replicating mRNA formulations (108). For example, a 5 *µ*g booster dose of the saRNA vaccine ARCT-154 elicited non-inferior neutralizing antibody titers against the ancestral SARS-CoV-2 strain and superior responses against the Omicron BA.4/5 variant compared to a 30 *µ*g dose of BNT162b2 (109). Seasonal influenza saRNA candidates, such as ARCT-2138 (110), have entered early-phase clinical evaluations, though public clinical data remain pending at the time of writing this manuscript. Additionally, the emerging circular RNA platform employs covalently closed single-stranded RNA molecules generated from linear precursors. This continuous structure confers resistance to exonucleolytic degradation and prolongs intracellular antigen translation, although clinical validation of this approach remains to be established (98, 111).

The mRNA technology platform offers several major advantages over traditional egg- and cell-culture-based SIV production platforms across manufacturing, antigenic match, and improvements in cell-mediated immunity. First, mRNA vaccines utilize a streamlined, highly reproducible manufacturing process that relies on standardized raw materials regardless of the target antigen sequence, enabling rapid scalability and eliminating reliance on a continuous egg supply (112–114). By replacing the sequence-encoding region within an established mRNA structural template, the manufacturing timeline from strain selection to vaccine release can be compressed to 2–3 months, compared to about six months typically required for conventional platforms (112, 114). This rapid turnaround can reduce the lag between WHO strain recommendations and seasonal deployment, allowing for strain selection closer to the onset of viral circulation and reducing the risk of pre-season antigenic drift (114). Furthermore, this agile production schedule creates the potential for finer geographical tailoring of vaccine formulations in seasons where predominant viral subclades diverge regionally across both Hemispheres (112). Second, the platform eliminates the need to propagate live influenza viruses in eggs or mammalian cell cultures, completely preventing cell- or egg-adaptive mutations, particularly for the A(H3N2) subtype (112–114). Indeed, Russell et al. (114) suggest that between 2011 and 2020, occurrence of egg-adaptive mutations was responsible for vaccine mismatch in six seasons, while antigenic drift caused vaccine mismatch in four seasons. Furthermore, because viral protein antigens are translated directly within human host cells *in vivo*, their primary structure, native conformation, and physiological glycosylation patterns are more preserved, thereby avoiding species-specific glycosylation discrepancies associated with avian or insect expression systems (112). As also mentioned earlier, differences in antigen glycosylation patterns can impair virus neutralization and therefore reduced effectiveness. Third, the inherent versatility of the platform simplifies multi-antigen polyvalency: in addition to surface glycoproteins (HA and NA), conserved internal influenza virus proteins can be added to confer broader cross-protection (112, 113). Analogously, incorporating antigens from other co-circulating respiratory viruses, such as SARS-CoV-2 and RSV, represents a viable approach to achieving a more comprehensive seasonal protection within a single formulation (114). Fourth, synthetic mRNA molecules and LNPs possess intrinsic immunostimulatory properties, triggering pattern recognition receptors to function as an adjuvant system (112, 115, 116). The intrinsic adjuvanticity of mRNA stems from its activation of multiple innate immune receptors, including endosomal TLRs 3, 7, and 8, as well as cytosolic retinoic acid-inducible gene I (RIG-I) and melanoma differentiation-associated protein 5 (MDA5). However, this attribute carries a dual role, as disproportionate adjuvanticity and dysregulated innate signaling can both reduce vaccine effectiveness and increase reactogenicity (116).

## 4 mRNA-1010 by Moderna

### 4.1 Original mRNA-1010 formulation

mRNA-1010 is a LNP-encapsulated vaccine developed by Moderna. The vaccine encodes the full-length, membrane-bound HA proteins of influenza strains recommended by the WHO for cell-based/recombinant vaccines (117). While early clinical development investigated quadrivalent formulations, later stages shifted to a trivalent formulation in alignment with WHO recommendations following the projected extinction of the B/Yamagata lineage (18, 19). To date, mRNA-1010 represents the most extensively evaluated seasonal influenza mRNA vaccine candidate in clinical trials (**Supplementary Table 1**).

In the first-in-human, phase I/II RCT 101 (117, 118), investigators evaluated the safety, humoral and cellular immunogenicity of varying mRNA dose levels (total HA content ranging from 6.25 *µ*g to 200 *µ*g) among adults aged ≥18 years. All evaluated formulations proved immunogenic and antibody responses were more robust in younger individuals than in older adults. When compared to sdIIV4, geometric mean titers (GMTs) measured via HAI assay trended higher for A(H1N1)pdm09 and A(H3N2), but this trend did not extend to both B strains. For the B strains, particularly B/Victoria, GMFRs from baseline to one month post-vaccination failed to consistently exceed a four-fold threshold and were comparable to or lower than those elicited by the comparator (**Figure 1A**). Conversely, for both A strains, mRNA-1010 elicited responses comparable to the sdIIV4 even at low doses of 6.25 *µ*g or 12.5 *µ*g. mRNA-1010 induced a strong polyfunctional CD4^+^ T-cell response that, interestingly, was more pronounced for influenza B. Furthermore, mRNA-1010 generated higher GMTs than the licensed comparator against three heterologous A(H3N2) strains, suggesting a capacity for broader cross-clade coverage. The safety profile was consistent with authorized mRNA vaccines against COVID-19. Dose-dependent reporting rates of solicited ARs were observed, with a more favorable reactogenicity profile noted at mRNA contents below 100 *µ*g. While mRNA-1010 was more reactogenic than the sdIIV4, showing approximately twice the reporting rate, the most common ARs (injection site pain, fatigue, myalgia, headache, and arthralgia) were predominantly mild-to-moderate and self-limiting. Based on these data, the mRNA-1010 formulation containing 12.5 *µ*g per strain (total HA content of 37.5 *µ*g and 50 *µ*g for trivalent and quadrivalent formulations, respectively) was selected for further clinical development (117, 118).

**Figure 1:**
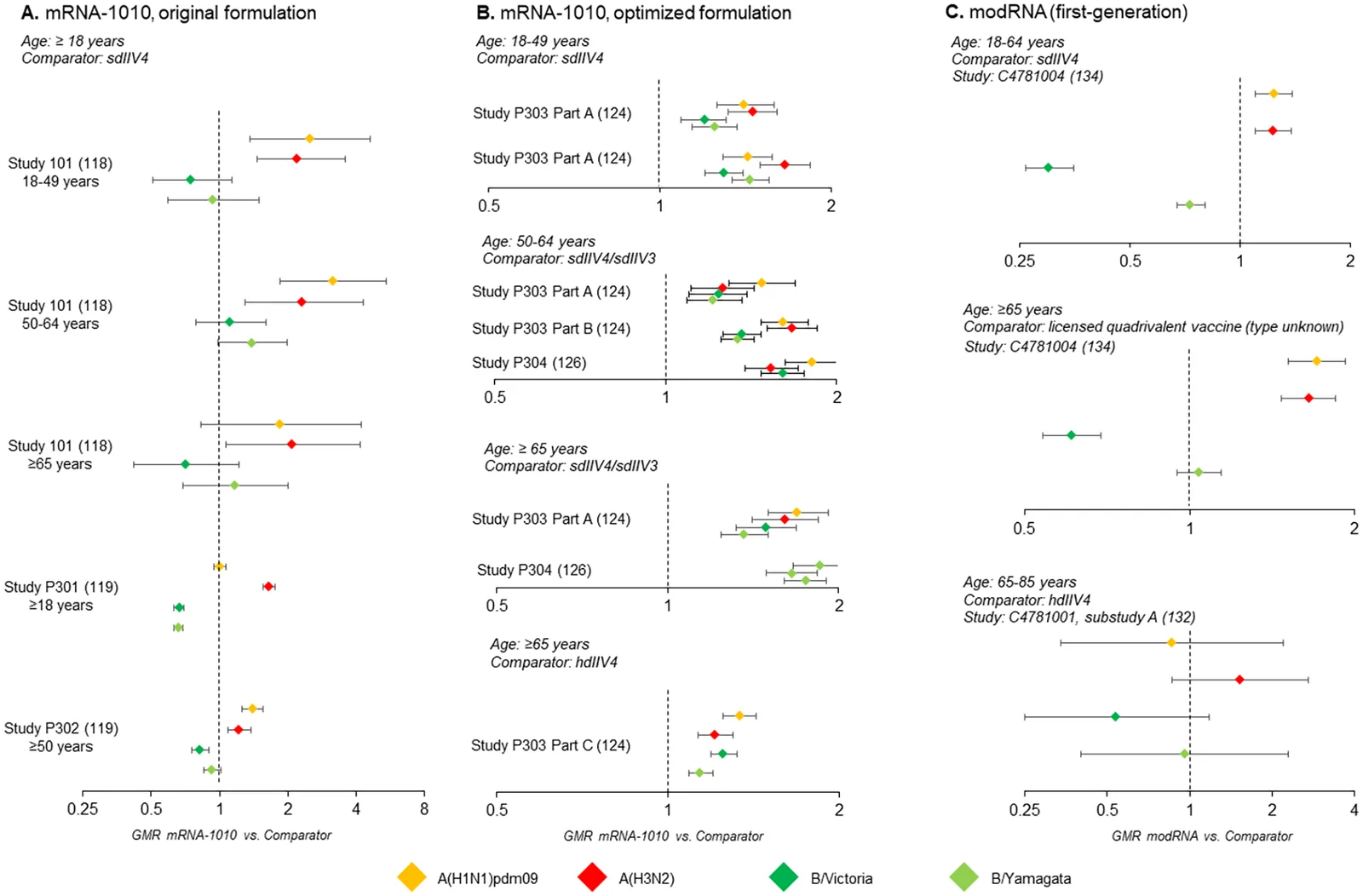
Post-vaccination geometric mean ratios of hemagglutination-inhibition antibody levels elicited by Moderna’s original **(A)** and optimized **(B)** mRNA-1010 formulations, and Pfizer’s first-generation modRNA candidate **(C)**, relative to egg-based split-virion influenza vaccines. Geometric mean ratios (GMRs) represent the ratio of geometric mean titers (GMTs) measured via hemagglutination-inhibition (HAI) assay one month post-vaccination following mRNA versus inactivated influenza vaccines. GMR values exceeding 1.0 indicate higher GMTs among subjects vaccinated with mRNA candidates. The original quadrivalent mRNA-1010 formulation (50 *µ*g total; 12.5 *µ*g per strain) was evaluated in the 101, P301, P302, and P303 trials, whereas the optimized trivalent mRNA-1010 formulation (37.5 *µ*g total; 12.5 *µ*g per strain) was used in the P304 study. Active comparators consisted of standard-dose quadrivalent egg-based inactivated split-virion influenza vaccines (sdIIV4) in the 101, P301, and P302 trials, a high-dose quadrivalent egg-based inactivated split-virion (hdIIV4) in the P303 older adult cohort, and either trivalent (sdIIV3) or quadrivalent standard-dose vaccines in the P304 study, depending on local availability. For the modRNA vaccine candidate, evaluated formulations were quadrivalent (30 *µ*g total; 7.5 *µ*g per strain) for younger adults. For older adults (≥65 years) in trial C4781004, the publicly posted ClinicalTrials.gov record identifies the investigational arm as “Dose Level 2” and the control as a licensed quadrivalent influenza vaccine; the publicly displayed intervention record does not provide the modRNA dose in micrograms or the comparator brand. Numeric values for certain GMR endpoints were extracted directly from ClinicalTrials.gov (134).

Two subsequent phase III RCTs evaluating the original 50 *µ*g mRNA-1010 formulation confirmed its insufficient immunogenicity against influenza B (119). The primary objectives of the P301 trial were to assess relative immunogenicity and safety, while rVE was the secondary objective; therefore, the P301 trial was not powered to detect differences in efficacy. Conversely, the co-primary endpoints of the P302 study were safety and rVE, whereas immunogenicity, assessed in a subset of participants, was the secondary endpoint. In the P301 study, the HAI response of mRNA-1010 compared to sdIIV4 was defined as non-inferior if a LL of the 97.5% CIs exceeded 0.667 (1.5-fold non-inferiority margin) for the geometric mean ratio (GMR) and exceeded –10% (10% non-inferiority margin) for the difference in seroconversion rates (ΔSCR). The non-inferiority criteria were successfully met for both A(H1N1)pdm09 and A(H3N2), but they were not achieved for both B/Victoria and B/Yamagata (**Figure 1A**). Consistent immunogenicity findings were observed within the immunogenicity subset of the P302 efficacy study. These immunogenicity outcomes directly reflected the rVE estimates. Consistent with the antibody data in the P301 study, the point rVE estimates of the original mRNA-1010 formulation versus sdIIV4 were 17.2% for influenza A but –66.0% for influenza B (**Table 1**). Importantly, the P301 trial confirmed that HAI titers function reliably as a correlate of protection for mRNA-based platforms; each one-standard-deviation increase in the day 29 HAI titer was associated with a 53%, 61%, and 52% reduction in the risk of influenza caused by A(H1N1)pdm09, A(H3N2), and B/Victoria, respectively. In the P302 study, the overall rVE was 1.7% against any influenza strain, 7.2% against influenza A, and –62.7% against influenza B (**Table 1**). However, the P302 trial had not accrued the pre-planned number of influenza cases used for the power calculations. Regarding safety endpoints, the incidence of any solicited AR within the first seven days post-vaccination was significantly higher in the mRNA-1010 cohorts (P301: 70.4%; P302: 71.5%) than in the active comparator groups (P301: 48.0%; P302: 50.4%). Conversely, the rates of unsolicited adverse events (AEs) within the first month post-vaccination remained comparable between the mRNA-1010 and comparator arms across both trials (P301: 12.1% vs. 13.3%; P302: 26.4% vs. 24.6%) (119).

**Table 1:** Relative vaccine efficacy of mRNA versus egg-based standard-dose split influenza vaccines.

Age, years	Virus	Original mRNA-1010 (50 *µ*g, quadrivalent) (119)	Optimized mRNA-1010 (37.5 *µ*g, trivalent) (125, 126)	modRNA (30 *µ*g, quadrivalent) (133, 134)
≥18	Influenza A	17.2 (–45.7, 53.3)^1^	–	–
	Influenza B	–66.0 (–187.9, 2.3)^1^	–	–
18–64	Any influenza	–	–	34.5 (7.4, 53.9)
≥50	Any influenza	1.7 (–24.1, 22.2)	26.6 (16.7, 35.4)	–
	Influenza A	7.2 (–18.8, 27.5)	26.5 (16.1, 35.5)	–
	A(H1N1)pdm09	0.3 (–45.4, 31.6)	29.6 (16.4, 40.7)	–
	A(H3N2)	16.7 (–18.9, 41.5)	22.2 (4.3, 36.9)	–
	Influenza B	–62.7 (–244.6, 23.1)	29.1 (–18.5, 57.5)	–
50–64	Any influenza	–	26.1 (12.3, 37.7)	–
	A(H1N1)pdm09	–	28.3 (9.5, 43.1)	–
	A(H3N2)	–	22.4 (–2.2, 41.1)	–
	Influenza B	–	23.0 (–54.8, 61.7)	–
≥65	Any influenza	–	27.4 (12.1, 40.0)	–5.8 (–47.2, 23.8)^2^
	A(H1N1)pdm09	–	31.2 (11.2, 46.7)	–
	A(H3N2)	–	22.0 (–7.1, 43.2)	–
	Influenza B	–	35.5 (–37.7, 69.8)	–

1 Relative efficacy was the secondary objective of the P301 study; therefore, it was not powered to detect differences in efficacy.

2 These data were reported in the publicly posted ClinicalTrials.gov results (134). For adults aged ≥65 years, the public intervention record identifies “Dose Level 2” and a licensed quadrivalent influenza vaccine comparator but does not state the modRNA dose in micrograms or the comparator brand.

SIVs are known to primarily drive a recall response by reactivating pre-existing memory B cells, which can contribute to “original antigenic sin” by preferentially boosting antibodies established during previous exposures (120). While antigen-stimulated B cells in draining lymph nodes rapidly differentiate into short-lived antibody-producing plasmablasts, a subset enters germinal center reactions where they undergo somatic hypermutation and affinity selection, ultimately yielding high-affinity long-lived bone marrow plasma cells and persistent memory B cells (121). Using ultrasound-guided fine-needle aspiration of lymph nodes alongside peripheral Ig sequencing to evaluate circulating antibody clonotype diversity and serum IgG proteomics, a recent study (122) demonstrated that mRNA-1010 elicited superior plasmablast frequencies, higher antibody titers, and markedly enhanced B-cell responses compared to sdIIV4. Importantly, mRNA-1010 induced persistent germinal center reactions lasting up to 26 weeks post-vaccination in a third (5/13) of participants, which was not observed among recipients of sdIIV4. Furthermore, mRNA-1010 elicited novel antibody clonotypes and expanded pre-existing clonotypes, translating into broader cross-reactive antibody binding against antigenically drifted A(H1N1) and A(H3N2) HA variants isolated over several decades. These results could stem from the intrinsic adjuvant properties of mRNA-based vaccines or extended antigen expression kinetics within draining lymph nodes that allow continuous engagement of germinal center B cells (122).

### 4.2 Optimized mRNA-1010 formulation

To address the suboptimal immunogenicity and efficacy against influenza B, Moderna structurally refined mRNA-1010 by introducing point mutations into non-antigenic HA regions, thereby optimizing antigen expression. Enhanced antigen expression and the consequent improvement in immunogenicity against both B strains were confirmed in a phase II study of healthy adults aged 18–49 years, which compared the safety and immunogenicity of the original formulation with two optimized versions of mRNA-1010 across various dose levels (123). While safety and tolerability profiles remained similar across all vaccine groups, the immune response against influenza B improved significantly with the optimized formulations. Specifically, compared to the original mRNA-1010, the optimized composition 1 (which differed from composition 2 by an updated mRNA manufacturing process) elicited much higher SCRs at a 50 *µ*g dose for both B/Victoria (36% vs. 13%) and B/Yamagata (50% vs. 29%) (123).

The optimized 50 *µ*g formulation was subsequently evaluated in the phase III P303 trial (124), which was conducted in three sequential parts. These parts varied by study population and active comparator: Part A enrolled adults aged ≥18 years using a sdIIV4 comparator; Part B enrolled younger adults aged 18–64 years using the same sdIIV4 comparator; and Part C evaluated older adults aged ≥65 years against hdIIV4. This optimization strategy proved successful. The optimized mRNA-1010 formulation met all prespecified non-inferiority and superiority criteria against both licensed comparators (**Figure 1B**). For instance, in Part B, mRNA-1010 elicited 32%–67% higher GMTs than the sdIIV4 across all four vaccine strains. Correspondingly, significantly higher SCR rates were achieved for A(H1N1)pdm09 (ΔSCR: 19.5%; 95% CI: 15.9%, 23.1%), A(H3N2) (ΔSCR: 22.5%; 95% CI: 18.9%, 26.0%), B/Victoria (ΔSCR: 14.7%; 95% CI: 11.5%, 17.8%), and B/Yamagata (ΔSCR: 12.2%; 95% CI: 8.9%, 15.5%). Among older adults enrolled in part C, mRNA-1010 was statistically superior to the hdIIV4 in terms of both GMR [A(H1N1)pdm09: 1.34 (95% CI: 1.25, 1.43); A(H3N2): 1.21 (95% CI: 1.13, 1.30); B/Victoria: 1.25 (95% CI: 1.19, 1.32); B/Yamagata: 1.14 (95% CI: 1.09, 1.20)] and ΔSCR [A(H1N1)pdm09: 13.4% (95% CI: 9.8%, 17.0%); A(H3N2): 8.6% (95% CI: 4.9%, 12.2%); B/Victoria: 9.7% (95% CI: 6.5%, 12.8%); B/Yamagata: 5.9% (95% CI: 2.8%, 9.0%). The reactogenicity and safety profiles of mRNA-1010 versus sdIIV4 in Parts A and B were consistent with observations from previous RCTs. When evaluated against the hdIIV4 in Part C, the incidences of both local (66.1% vs. 38.9%) and systemic (61.3% vs. 32.9%) solicited ARs were higher among recipients of mRNA-1010 (**Figure 2A**). Conversely, the rates of unsolicited AEs reported within four weeks post-vaccination remained comparable between the study arms (10.3% vs. 9.2%). SAEs were uncommon in both cohorts, occurring at rates of 0.6% and 0.3% for mRNA-1010 and hdIIV4, respectively (124).

**Figure 2:**
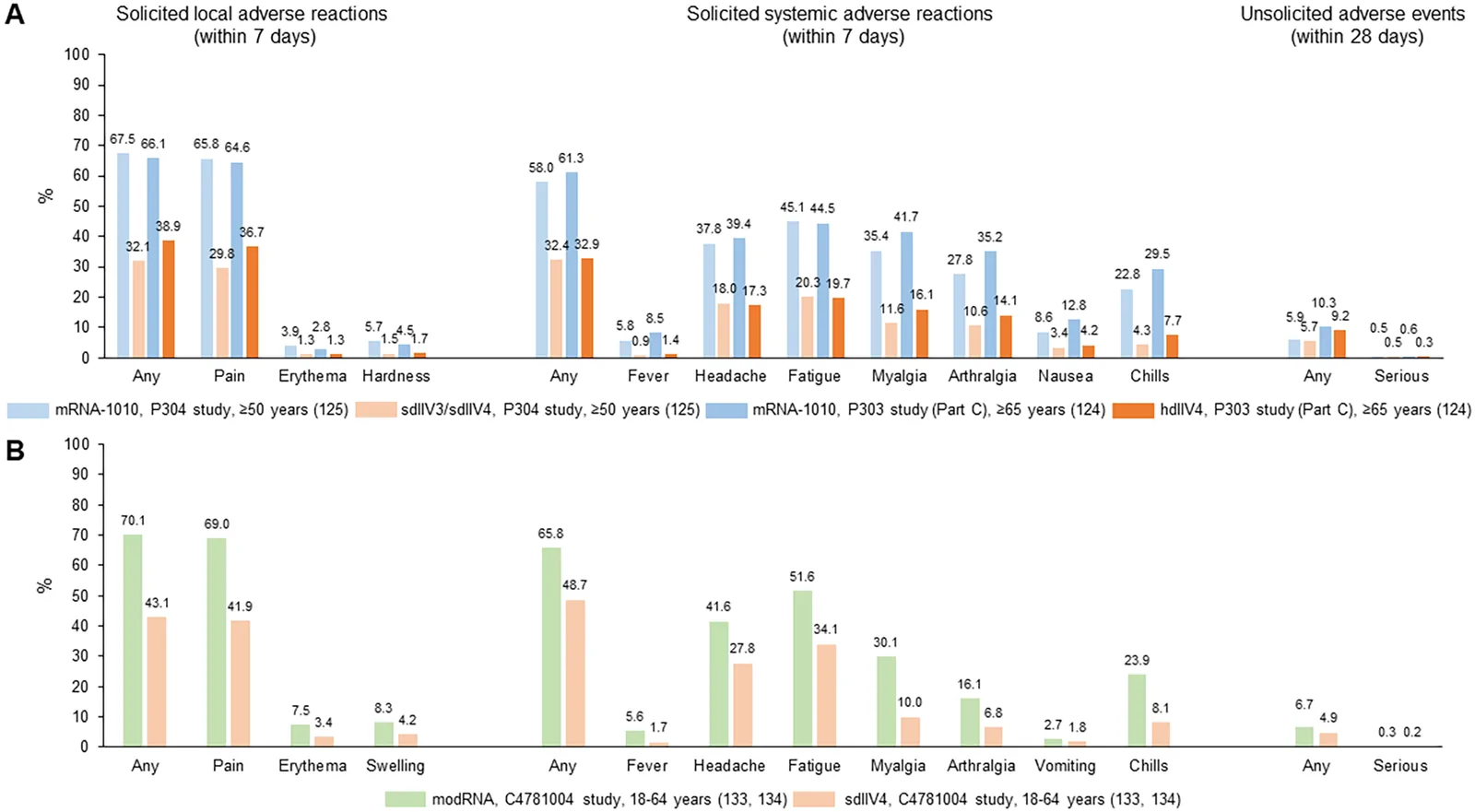
Frequency of solicited adverse reactions and unsolicited adverse events following a single dose of mRNA-1010 **(A)** or modRNA **(B)** vaccines compared to egg-based split-virion influenza vaccines. Frequency of events is based on findings from phase III randomized controlled trials. The optimized trivalent mRNA-1010 formulation (37.5 *µ*g total; 12.5 *µ*g per strain) was evaluated in the P304 trial, while the original quadrivalent formulation (50 *µ*g total; 12.5 *µ*g per strain) was used in the P303 study. For the first-generation modRNA vaccine candidate, the evaluated formulation was quadrivalent (30 *µ*g total; 7.5 *µ*g per strain).

The immunogenicity advantages of the optimized mRNA-1010 were subsequently confirmed in the pivotal phase III efficacy trial P304, conducted across 11 Northern Hemisphere countries during the 2024/2025 season (125). In this study, a total of 40,805 adults aged ≥50 years were randomized 1:1 to receive either a single dose of the trivalent mRNA-1010 formulation (37.5 *µ*g total; 12.5 *µ*g per strain) or a licensed comparator (sdIIV3 or sdIIV4, depending on local availability). The entire cohort underwent active surveillance for new-onset or worsening respiratory illness. Participants presenting with symptoms were evaluated via nasopharyngeal swab and tested using reverse-transcription polymerase chain reaction (RT-PCR) to confirm influenza infection. The primary endpoint was rVE against laboratory-confirmed influenza-like illness (ILI) occurring ≥14 days post-vaccination. Protocol-defined ILI required the presence of ≥1 systemic symptom (temperature >37.2 °C, chills, feverishness, tiredness, headache, or myalgia) and ≥1 respiratory symptom (sore throat, cough, sputum production, wheezing, or difficulty breathing). Success criteria were evaluated sequentially. First, non-inferiority was assessed, requiring the LL of the 95% CI for the rVE of mRNA-1010 versus the comparator to exceed –10%. If non-inferiority was established, the rVE was sequentially evaluated for superiority (defined as a 95% CI LL >0%) and, subsequently, higher-level superiority (defined as a 95% CI LL >9.1%). Within the entire cohort, laboratory-confirmed ILI was detected in 2.0% (411/20,179) of participants in the mRNA-1010 arm compared to 2.8% (557/20,124) of those in the comparator arm. This translated to an overall rVE of 26.6% (95% CI: 16.7%, 35.4%) against any influenza strain, thereby successfully meeting all three hierarchical success criteria. An analysis stratified by virus (sub)type showed consistency, with rVE estimates ranging from 22.2% for A(H3N2) to 29.6% for A(H1N1)pdm09. Efficacy estimates also remained robust when analyzed by age group, yielding a rVE of 26.1% among younger adults (50–64 years) and 27.4% among older adults (≥65 years) (**Table 1**). Furthermore, exploratory subgroup analyses consistently favored mRNA-1010, demonstrating stable rVE point estimates when stratified by sex, race, body mass index, baseline risk status, frailty index, previous-season vaccination status, geographic region, and the type of comparator received (sdIIV3 or sdIIV4) (125, 126).

Within the immunogenicity subset (approximately 6% of the cohort) of the pivotal P304 trial, mRNA-1010 demonstrated superior immunogenicity compared to the licensed active comparators across both HAI and microneutralization assays. In the HAI assay, point GMR estimates were 53%–81% higher among adults aged 50–64 years (LLs of the 95% CIs exceeding 1.3) and 65%–85% higher among older adults aged ≥65 years (LLs of the 95% CIs exceeding 1.4) (**Figure 1B**). Similarly, GMRs for neutralizing antibodies consistently favored mRNA-1010 in both the 50–64 age bracket (GMR range: 1.5–3.0) and the older adult cohort (GMR range: 1.3–2.7) (125, 126).

Safety analyses from the P304 trial confirmed expected reactogenicity and safety profiles (**Figure 2A**). Overall, solicited ARs were reported at approximately twice the rate among recipients of mRNA-1010 compared to the licensed products. Among individuals vaccinated with mRNA-1010, injection-site pain was the most prevalent local AR (67.5%), whereas fatigue (45.1%), headache (37.8%), and myalgia (35.4%) constituted the most common systemic ARs. The majority of these reactions were mild-to-moderate in severity, with a median duration of 1–2 days. Unsolicited AEs within one month post-vaccination occurred at comparable rates (5.9% for mRNA-1010 vs. 5.7% for the comparator), while SAEs were equally distributed at 0.5% in both study arms (125, 126).

Additionally, an open-label phase I RCT compared the original quadrivalent mRNA-1010 vaccine the quadrivalent aIIV (aIIV4), using a systems biology approach across various dimensions of humoral and cell-mediated immunity (127–129). One month post-vaccination, point HAI GMFR estimates in the mRNA-1010 cohort were slightly lower than those in the aIIV4 group for A(H1N1)pdm09 (4.9 vs. 5.9), A(H3N2) (3.7 vs. 4.5), and B/Victoria (1.5 vs. 2.0), but were identical for B/Yamagata (2.6 in both arms). Correspondingly, SCRs remained comparable between the two vaccine platforms for A(H1N1)pdm09 (67.3% vs. 61.4%), A(H3N2) (54.5% vs. 59.6%), and B/Yamagata (34.5% vs. 35.1%), but diverged significantly for B/Victoria, which favored the adjuvanted comparator (9.1% vs. 35.1%). In contrast to the standard serology, mRNA-1010 generally elicited a more pronounced increase in systemic cytokine and chemokine levels, including interferon-*γ*, interleukin-6, and interleukin-2 receptor alpha (127). From day 29 through day 361, antibody levels declined at a similar rate for both vaccines while remaining consistently above baseline pre-vaccination levels. Both formulations similarly enhanced HA-specific memory B-cell responses against A(H1N1)pdm09 and both influenza B strains; however, mRNA-1010 induced a higher frequency of memory B cells specific to the A(H3N2) component. While the vaccines elicited comparable CD4^+^ T-cell responses, mRNA-1010 demonstrated a distinct trend toward higher CD8^+^ T-cell responses targeted specifically against both influenza B strains, though not against the influenza A strains (128). A closer evaluation of the humoral immune response revealed a distinct functional profile for mRNA-1010. Specifically, compared to aIIV4, mRNA-1010 elicited more rapid and complete maturation of IgG1, IgA, and Fc-receptor binding, broader HA-specific binding antibody profiles, enhanced natural killer (NK) cell activation, and a higher frequency of HA-specific, neutrophil-activating antibodies (129).

In August 2026, the optimized trivalent mRNA-1010 vaccine (12.5 *µ*g of mRNA per strain) was approved in the US for adults aged ≥50 years (130).

## 5 modRNA by Pfizer

modRNA is a nucleoside-modified mRNA influenza vaccine candidate developed by Pfizer that encodes full-length HA from selected influenza strains. It uses Pfizer’s nucleoside-modified mRNA–LNP platform; public sources support platform commonality with BNT162b2 but do not establish that the investigational influenza vaccine uses an identical LNP formulation. Preclinical evaluation of monovalent, trivalent, and quadrivalent formulations of modRNA demonstrated an ability to elicit robust, durable humoral and cell-mediated responses with an acceptable safety profile in both mouse and non-human primate models (131). The subsequent first-in-human RCT (C4781001) (132) comprised a phase I substudy A, which enrolled older adults aged 65–85 years, and a phase I/II substudy B. Substudy B used an initial and expanded enrollment scheme spanning both older (65–85 years) and younger (18–64 years) cohorts to determine the optimal dose levels for subsequent phase III evaluation. In substudy A, participants were randomized into 14 cohorts (approximately 15 per investigational group) to receive various formulations of monovalent, bivalent, or quadrivalent modRNA vaccines with total modRNA content ranging from 3.75 *µ*g to 30 *µ*g or hdIIV4 comparator (**Supplementary Table 1**). In substudy A, solicited ARs increased in a dose-dependent manner. Post-vaccination HAI GMTs increased with a dose-dependent trend; the increase was much higher for influenza A strains than for influenza B strains. In the subsequent substudy B, 349 older adults received either modRNA 30 *µ*g, modRNA 60 *µ*g, or hdIIV4, while 265 younger adults were randomized to receive either modRNA 30 *µ*g or modRNA 60 *µ*g. Across both age cohorts, pain (44.3%–62.6%) was the most common local solicited AR, while fatigue (33.0%–43.5%), headache (20.9%–38.2%), and myalgia (16.5%–32.1%) were the most frequent systemic ARs. Most ARs were mild-to-moderate in severity and resolved within 1–2 days. While reactogenicity was generally reported more frequently in both modRNA arms compared to hdIIV4, unsolicited AEs were distributed similarly across groups (6.9%–8.5% for modRNA and 7.0% for hdIIV4). In older adults, one-month post-vaccination GMFRs were numerically higher in the modRNA 60 *µ*g arm than in the hdIIV4 arm for A(H1N1)pdm09 (4.9 vs. 4.1) and A(H3N2) (3.5 vs. 2.3), but lower for B/Victoria (1.7 vs. 2.7) and B/Yamagata (1.4 vs. 2.8). In younger adults, both the 30 *µ*g and 60 *µ*g doses showed similar GMFRs. A comparable pattern was observed for SCRs. In contrast, evaluation of cellular immunogenicity one week after immunization showed overall higher CD4^+^ and CD8^+^ responses against all four strains with modRNA compared to hdIIV4, and T-cell responses were similar or higher following the 60 *µ*g dose compared to the 30 *µ*g dose. Consequently, the authors proposed selecting modRNA 60 *µ*g for older adults and modRNA 30 *µ*g for younger adults in the subsequent phase III study (132).

Study C4781004 (133, 134) was a phase III trial with the primary objective of evaluating the rVE of modRNA versus sdIIV4 against RT-PCR-confirmed ILI, which was defined similarly to the previously described mRNA-1010 efficacy trial. The trial enrolled both younger (18–64 years) and older (≥65 years) adults. However, as of time of writing, only data for the younger cohort were published (133), while certain data for the older cohort were available only on the trial registry (134) and therefore remain subject to peer review. Among younger adults, the quadrivalent modRNA candidate was administered at a total dose of 30 *µ*g. In older adults, the publicly posted ClinicalTrials.gov (134) results identify the experimental vaccine as “Dose Level 2” and the comparator as a licensed quadrivalent influenza vaccine; the publicly displayed intervention record does not state the modRNA dose in micrograms or identify the comparator brand. During the 2022/2023 influenza season, most cases were caused by influenza A strains. The rVE among 18–64-year-olds was 34.5% (95% CI: 7.4%, 53.9%), meeting both non-inferiority and superiority criteria (133). Conversely, in adults aged ≥65 years, the publicly posted ClinicalTrials.gov (134) results report a rVE point estimate of –5.8% (95% CI: –47.2%, 23.8%); the pre-specified non-inferiority criterion was not met. Publicly available results do not establish the reason for this outcome.

In the immunogenicity subset, GMRs of modRNA versus the comparator proved non-inferior and numerically superior for both A strains across both age cohorts (with LLs of the 95% CIs exceeding the pre-specified non-inferiority margin of 0.67); however, non-inferiority was not achieved for B/Yamagata and especially B/Victoria (**Figure 1C**). Conversely, modRNA demonstrated a distinct advantage in eliciting cellular immunity: increases in CD4^+^ and CD8^+^ T-cell frequencies were higher in the modRNA group across all strains from one week through six months post-vaccination, a trend that was particularly evident for CD8^+^ T-cell expression against both B strains (133).

As expected, the modRNA candidate was more reactogenic than sdIIV4 in terms of both injection-site (70.1% vs. 43.1%) and systemic (65.8% vs. 48.7%) solicited ARs. Pain (69.0%), fatigue (51.6%), and headache (41.6%) constituted the three most common ARs, the majority of which were mild-to-moderate in severity and self-limiting within 2 days. Unsolicited AEs reported within the first month post-vaccination were more balanced between study arms (6.7% in the modRNA group vs. 4.9% in the control group), while the frequency of SAEs remained very low in both cohorts (**Figure 2B**) (133).

Besides multivalent modRNA formulations, a human challenge trial evaluating a monovalent A(H1N1)pdm09-based modRNA vaccine (7.5 *µ*g) is of particular interest (135). Such RCTs are highly valuable during clinical development, as they enable the assessment of vaccine efficacy in a strictly controlled environment. In this Phase IIa study, healthy, challenge-virus-naïve adults aged 18–55 years were randomized to receive either modRNA or sdIIV4. An unvaccinated control group was sourced from a separate trial in which subjects were challenged with the identical virus. Intranasal challenge was performed one month post-vaccination. From day 1 to day 8 post-challenge, RT-PCR-confirmed symptomatic influenza (grade ≥2) was diagnosed in 24.2% of the control group, 0% of the modRNA group (absolute efficacy: 100%; 95% CI: 75.2%, 100%), and 4.2% of the sdIIV4 group (absolute efficacy: 84.5%; 95% CI: 43.4%, 96.0%). No febrile influenza occurred in either vaccinated group (absolute efficacy: 100%), compared to an incidence of 17.3% in the control group. Viral shedding was analogously reduced among all vaccinees (135).

Similar to Moderna, Pfizer initiated the development of a second-generation modRNA influenza vaccine (136, 137). However, as of writing this manuscript, no results have been published in the peer-reviewed literature.

## 6 Other mRNA seasonal vaccine candidates tested in randomized controlled trials

A monovalent A(H1N1)pdm09 nucleoside-modified mRNA vaccine candidate developed by GSK/CureVac (FLUmHA) was evaluated in a proof-of-concept phase I study (138). Younger (18–45 years) and older (60–80 years) adults were randomized to receive either FLUmHA (doses ranging from 0.5 to 100 *μ*g in younger adults, or 18 *μ*g in older adults) or sdIIV4. In younger adults, 7-day solicited ARs showed a dose-dependent increase, ranging from 62.5% at low doses (0.5–6 *μ*g) to 100% at high doses (54–100 *μ*g), compared to 88.6% for sdIIV4. In older adults, 62.5% of FLUmHA recipients and 56.3% of sdIIV4 recipients reported ≥1 AR. Mild-to-moderate pain and fatigue were the most frequent local and systemic ARs, respectively. Unsolicited AEs within the first month post-vaccination demonstrated a similar dose-dependent trend. Of five SAEs reported during six-month follow-up, none were vaccine-related. In younger adults, HAI GMFRs from baseline to day 22 increased with rising dose levels, ranging from 8.6 at 0.5 *μ*g to 29.6 at 100 *μ*g, compared to 10.0 for sdIIV4. Consequently, the HAI response elicited by FLUmHA was statistically superior to sdIIV4 at doses ≥18 *μ*g. In older adults, FLUmHA at 18 *μ*g was also more immunogenic than sdIIV4, achieving a GMR of 2.33. Although HAI titers declined over time, they remained above baseline levels for up to six months post-vaccination. In both age cohorts, FLUmHA elicited numerically higher HA-specific CD4^+^ T-cell responses than sdIIV4 (138).

Given the high rate of antigenic drift within the influenza A(H3N2), observational studies have consistently shown that vaccine effectiveness is lower for this subtype relative to other seasonal strains, especially among older adults (139). Consequently, incorporating additional A(H3N2) strains, also to replace B/Yamagata represents a promising strategy to broaden coverage against co-circulating clades, mitigate the risk of vaccine mismatch, and ultimately improve the overall vaccine effectiveness, provided that significant immunological interference does not occur. To evaluate this approach, Moderna assessed three next-generation seasonal mRNA influenza vaccines [pentavalent mRNA-1011.1 with an additional A(H3N2) strain; quadrivalent mRNA-1011.2, in which B/Yamagata was replaced with an additional A(H3N2) strain; and pentavalent mRNA-1012 with two additional A(H3N2) strains and no B/Yamagata] against the original quadrivalent mRNA-1010 in healthy adults aged 50–75 years (140). These investigational formulations demonstrated safety and reactogenicity profiles comparable to mRNA-1010, with no vaccine-related SAEs reported. All three next-generation candidates elicited robust HAI antibody responses that were non-inferior to mRNA-1010 for matched strains, while generating significantly higher titers against the newly added A(H3N2) strains. Importantly, the immunogenicity of the A(H1N1)pdm09 and influenza B components was preserved, while the omission of B/Yamagata did not impact responses to B/Victoria (140).

All mRNA vaccines discussed so far targeted HA alone. Historically, the clinical development of SIVs has predominantly focused on humoral responses against HA, for which a correlate of protection has been established, while NA, the second major surface glycoprotein of influenza A and B, has been largely neglected. Indeed, even in most currently licensed SIVs, NA antigen quantity and quality vary and are not standardized. Anti-NA antibodies are known to limit viral egress, thereby lessening disease severity and reducing viral shedding (141, 142). Evidence also suggests that despite weak-to-moderate correlation between anti-NA and HAI or neutralizing antibody titers, anti-NA antibodies may act as an independent correlate of protection (143). To improve vaccine effectiveness, Moderna evaluated two next-generation quadrivalent candidates that simultaneously encode both HA and NA. In that first-in-human RCT (144), mRNA-1020 (HA: NA ratio 1:1) and mRNA-1030 (HA: NA ratio ≈ 3:1) were tested at total antigen doses ranging from 50 *μ*g to 150 *μ*g. The original HA-only mRNA-1010 and the licensed NA-free RIV4 served as comparators. Both dual-antigen mRNA formulations elicited robust HA antibody responses comparable to both control vaccines, while successfully inducing strong, NA-specific antibody responses without increasing mRNA-associated reactogenicity at equivalent dose levels (144).

## 7 Seasonal influenza plus COVID-19 combination vaccines

Combination vaccines offer several advantages, including a reduced cumulative number of injections and clinical visits, improved compliance with lower rates of missed opportunities for vaccination, enhanced distribution efficiency through a reduced cold-chain capacity requirement and lower administration costs (145). In recent years, Moderna evaluated both first-generation (mRNA-1073) and second-generation (mRNA-1083) combination vaccine candidates targeting seasonal influenza and COVID-19. The first-generation construct, mRNA-1073, combined the original influenza candidate mRNA-1010 with the licensed monovalent mRNA-1273 vaccine encoding the spike (S) glycoprotein of ancestral SARS-CoV-2 (146). Building upon this, the second-generation candidate mRNA-1083, which was approved in Europe for adults aged ≥50 years (147), combines an optimized mRNA-1010 formulation with mRNA-1283. The latter is a next-generation antigen encoding two subdomains of the SARS-CoV-2 S protein, specifically the *N*-terminal domain (NTD) and the receptor-binding domain (RBD) (146, 148). Similar to the developmental path of mRNA-1010, the available RCTs evaluating both combination candidates utilized quadrivalent SIV components, as trial protocols were initiated prior to official public health recommendations calling for the removal of the B/Yamagata lineage (146, 148–150). The authorized mRNA-1083 formulation contains approximately 31.6 *μ*g of total mRNA (8.3 *μ*g for each of the three seasonal influenza strains and 6.7 *μ*g for SARS-CoV-2) per single 0.32 ml dose in a pre-filled syringe (147).

In a phase I/II RCT evaluating the first-generation combination candidate mRNA-1073 in healthy adults aged 18–75 years (146), the vaccine showed dose-dependent reactogenicity. Local and systemic solicited ARs were lower in the 25 *μ*g and 50 *μ*g dose groups compared to the 100 *μ*g cohort. Notably, reactogenicity profiles following administration of the 25 *μ*g and 50 *μ*g doses of mRNA-1073 were lower than those observed in participants who received co-administered single components (mRNA-1010/50 *μ*g plus mRNA-1273/50 *μ*g). No vaccine-related SAEs were reported. All evaluated doses of mRNA-1073 elicited robust and durable humoral immune responses through six months post-vaccination against SARS-CoV-2 and all four matched influenza strains. However, post-vaccination HAI GMTs for influenza strains and geometric mean concentrations (GMCs) of anti-S antibodies measured via pseudovirus neutralization assay (psVNA) were numerically lower across all mRNA-1073 dose levels than those induced by separate component administration or co-administration of mRNA-1010 plus mRNA-1273 (146).

In the first-in-human phase I/II study of the second-generation combination vaccine mRNA-1083, younger (18–64 years) and older adults (65–79 years) were randomized into 12 study cohorts per age group (148). Participants received various mRNA-1083 formulations differing in total mRNA content (from 15 *μ*g to 82 *μ*g total mRNA in younger adults, and 27.5 *μ*g to 82 *μ*g in older adults) and mRNA ratios of total influenza HA to SARS-CoV-2 components (from 1:5 to 1:20). These were evaluated against active comparators, including the original and optimized quadrivalent mRNA-1010 seasonal influenza formulations (50 *μ*g), bivalent COVID-19 mRNA vaccines (mRNA-1283/10 *μ*g and mRNA-1273/50 *μ*g), and licensed influenza vaccines (sdIIV4 across both age cohorts, and hdIIV4 in older adults). Although most solicited ARs were mild-to-moderate in severity, their overall frequency was higher across mRNA-1083 dose arms relative to licensed stand-alone influenza or COVID-19 comparators. Unsolicited AEs were comparable between all treatment groups. At one month post-vaccination, mRNA-1083 elicited HAI GMTs against all four influenza strains, as well as anti-S psVNA GMCs against SARS-CoV-2, that were comparable to or numerically higher than those induced by licensed influenza and SARS-CoV-2 comparator vaccines (148).

A formulation of mRNA-1083 with a total mRNA content of 40 *μ*g advanced to pivotal phase III evaluation (149). The trial comprised two substudies, each characterized by independent pre-specified hypotheses. The first substudy enrolled adults aged 50–64 years randomized to receive either mRNA-1083 plus placebo or co-administered mRNA-1273 plus sdIIV4. The second substudy randomized older adults aged ≥65 years to receive either mRNA-1083 plus placebo or co-administered mRNA-1273 plus hdIIV4. Non-inferior immunogenicity of mRNA-1083 was successfully demonstrated for all four influenza strains and SARS-CoV-2 across both age cohorts. Specifically, at one month post-vaccination, the LLs of the 97.5% CIs for GMRs of mRNA-1083 versus the co-administration control groups were well above the non-inferiority margin of 0.667, while ΔSCRs largely exceeded the pre-specified –10% margin. In secondary superiority analyses involving the younger cohort, mRNA-1083 induced significantly higher antibody responses than co-administered mRNA-1273 plus sdIIV4 for all five viral targets, with GMRs ranging from 1.154 (95% CI: 1.115, 1.195) for B/Yamagata to 1.414 (95% CI: 1.333, 1.500) for A(H1N1)pdm09; ΔSCRs demonstrated concordant superiority patterns. Among older adults, mRNA-1083 demonstrated statistical superiority over co-administered mRNA-1273 plus hdIIV4 for HAI antibody responses against A(H1N1)pdm09 [GMR: 1.155 (95% CI: 1.094, 1.220); ΔSCR: 5.4% (95% CI: 2.4%, 8.4%)], A(H3N2) [GMR: 1.063 (95% CI: 1.007, 1.122); ΔSCR: 4.0% (95% CI: 1.0%, 7.1%)], and B/Victoria [GMR: 1.118 (95% CI: 1.070, 1.167); ΔSCR: 4.5% (95% CI: 1.9%, 7.2%)], as well as for anti-S neutralizing responses against the Omicron XBB.1.5 SARS-CoV-2 strain [GMR: 1.641 (95% CI: 1.526, 1.765); ΔSCR: 12.8% (95% CI: 10.0%, 15.5%)]. Superiority was not met for the B/Yamagata lineage in this older cohort [GMR: 1.007 (95% CI: 0.973, 1.042); ΔSCR: –1.4% (95% CI: –3.3%, 0.4%)] (149). At six months post-vaccination, antibody levels remained higher in the mRNA-1083 group than in the co-administration control groups across both age cohorts for A(H1N1)pdm09 (by 27.3% in adults aged 50–64 years and 20.6% in adults aged ≥65 years), A(H3N2) (by 32.2% and 13.5%, respectively), and SARS-CoV-2 (by 31.1% and 47.3%, respectively) (150). In both age cohorts, rates of any solicited ARs, the vast majority of which were mild-to-moderate in severity, were only slightly higher in the mRNA-1083 arm (85.2% in adults aged 50–64 years; 83.5% in adults aged ≥65 years) than in the co-administration control arms (81.8% for mRNA-1273 plus sdIIV4; 78.1% for mRNA-1273 plus hdIIV4, respectively). Among mRNA-1083 recipients, injection-site pain was the most common local AR (77.9% and 73.4% across the younger and older cohorts, respectively). The most frequent systemic ARs across both age groups were fatigue (58.9% and 52.8%), myalgia (58.3% and 51.1%), headache (51.4% and 43.5%), and arthralgia (47.0% and 42.2%) (149). Unsolicited AE rates were balanced between the mRNA-1083 and co-administration control groups in both younger and older cohorts through one month (9.7% vs. 9.4% and 11.9% vs. 12.2%, respectively) (149) and six months post-vaccination (18.9% vs. 18.8% and 25.0% vs. 24.9%, respectively) (150).

In a similarly designed phase III trial in Japanese adults aged ≥50 years, mRNA-1083 (40 *μ*g) met primary non-inferiority and secondary superiority criteria against all four influenza strains and SARS-CoV-2 JN.1 at one month post-vaccination, compared to co-administration of mRNA-1273 and a locally licensed sdIIV4. At sex months post-vaccination, HAI titers remained comparable to or numerically higher than those elicited by the active comparators. No treatment-related SAEs or deaths were reported (151).

A combination mRNA vaccine candidate co-targeting seasonal influenza and COVID-19 is currently under clinical evaluation by Pfizer/BioNTech (136, 152), though peer-reviewed trial results remain unpublished as of time of writing.

## 8 Concluding remarks

The clinical development of mRNA platforms, highlighted by the recent European authorization of the combination mRNA-1083 vaccine (147) and US approval of mRNA-1010 (130), represents a transformative shift toward overcoming some limitations of traditional SIVs. By eliminating reliance on viral propagation in eggs or cell cultures, mRNA technology preserves exact primary sequence identity with WHO-selected reference strains, ensuring the target antigen avoids structural modifications. Regarding the preferred product characteristics outlined by the WHO (14), accelerating manufacturing timelines to under five months from strain selection to finished product appears technically achievable. However, the real-world utility of this rapid production capability remains constrained by existing regulatory frameworks, requiring coordinated efforts among the WHO, regulatory agencies, and vaccine manufacturers to establish later strain selection timelines (114).

Standalone and combination mRNA seasonal influenza vaccines represent viable alternatives to currently licensed formulations. Across available RCTs, evaluating predominantly mRNA-1010, mRNA platforms induced robust and durable immune responses against all vaccine-like strains. Notably, this humoral immunogenicity exceeded that of sdIIVs (124, 125) and, in a few direct comparisons, some enhanced formulations (124), though comparative immunogenicity data against hdIIV, aIIV, and RIV remain sparse. This immunogenic advantage was particularly pronounced against influenza A strains, which account for the majority of adult influenza morbidity and mortality (153, 154). Emerging evidence suggests these heightened responses are underpinned by unique B-cell dynamics. Extended germinal center reactions induced by mRNA platforms permit continuous somatic hypermutation, expanding serological IgG diversity through both the generation of novel clonotypes and intraclonal expansion (122). Furthermore, mRNA vaccines drive distinct Fab and Fc functional profiles, accelerating antibody maturation toward IgG1 and IgA subclasses with enhanced Fc-receptor binding capable of augmenting antibody-dependent NK cell activation (129). Importantly, this sustained germinal center activity suggests that the vaccine-induced humoral responses are not substantially impeded by pre-existing immunity (122). Prior-season immunization among repeated vaccinees can reduce antibody-affinity maturation to the HA1 domain of A(H1N1)pdm09, A(H3N2), and B strains, a phenomenon that may contribute to reduced vaccine effectiveness (155). Although several meta-analyses (156, 157) indicate that prior-season receipt of conventional SIVs can attenuate current-season effectiveness, the real-world impact of repeated vaccination is highly complex and varies across target populations, epidemic seasons, antigenic distances between circulating and vaccine strains, and age-specific immunological landscapes (158). Nevertheless, systematic evidence consistently supports annual revaccination, reinforcing that receiving SIV in two consecutive years provides superior protection compared to receiving no vaccine (156, 157).

A key challenge highlighted by RCTs of both the original mRNA-1010 and modRNA formulations is their suboptimal humoral immunogenicity against influenza B lineages, particularly failing to achieve statistical non-inferiority compared to sdIIVs (119, 132, 133). Discrepancies between influenza A and B vaccine-induced immunity are well-documented across multiple platform technologies, with influenza B strains generally eliciting less robust responses. Mechanistically, this reduced immunogenicity may stem from complex host factors, such as differential immunological imprinting and prior exposure history, as well as the inherent immunogenic properties of influenza B surface antigens (119, 159). Furthermore, the HAI assay has lower sensitivity and higher variability for influenza B than for influenza A, often requiring ether treatment that can compromise assay specificity (159). HAI assay interpretation for influenza B is further confounded by age, pre-existing antibody levels, and lineage-specific dynamics (132). Overcoming suboptimal immunogenicity against influenza B requires targeted platform engineering, such as introducing stabilizing point mutations, tuning 5′ and 3′ UTRs, and refining LNP tropism, to systematically enhance antigen expression, processing, and durability. For example, to overcome initial B-strain limitations, the optimized mRNA-1010 formulation (now US-approved) incorporated a structurally refined influenza B HA encoding stabilizing point mutations in non-surface-exposed regions, thereby improving antigen expression without altering antigenic epitopes. This structural optimization translated directly into improved immunogenicity against B/Victoria and B/Yamagata compared to both sdIIV4 and hdIIV4 (124).

Cellular immune profiling reveals some distinct characteristics of mRNA platforms. mRNA candidates generate robust CD4^+^ T-cell expansion exceeding that observed with sdIIVs (133) and achieving levels comparable to adjuvanted formulations (128), providing helper T-cell activity that is essential for mitigating severe disease outcomes in vulnerable cohorts (160). Conversely, data from mRNA-1010 demonstrate inconsistent CD8^+^ T-cell induction (118). Cytotoxic CD8^+^ T cells play a critical role in controlling viral replication in the absence of neutralizing antibodies and driving rapid clinical recovery through cross-reactive epitopes (161). However, this relative deficiency in CD8^+^ engagement represents a shared limitation with current SIVs, which historically induce weak CD8^+^ activity (162) regardless of whether subunit sdIIV3, split sdIIV3, or aIIV3 formulations are administered (163).

Whether the heightened immunogenicity of mRNA platforms translates into clinically meaningful reductions in laboratory-confirmed influenza illness has been evaluated across three large-scale rVE trials (119, 125, 133). Initial data from the original mRNA-1010 formulation failed to demonstrate non-inferiority relative to sdIIV4 among adults aged ≥50 years (rVE 1.7%), an outcome attributed by the sponsor to lower-than-projected case accrual (119). However, an optimized mRNA-1010 formulation subsequently met all primary endpoints, achieving higher-level superiority over sdIIV with a rVE of 26.6% in this population (125). Similarly, Pfizer’s first-generation quadrivalent modRNA candidate demonstrated 34.5% rVE versus a licensed sdIIV4 among adults aged 18–64 years during an influenza A-predominant season (133). Nevertheless, in adults aged ≥65 years, the pre-specified non-inferiority criterion was not met (rVE –5.8%; 95% CI: –47.2%, 23.8%) (134, 164). The wide CI indicates imprecision and should not be interpreted as proving no clinical advantage. Whether second-generation modRNA constructs will improve protection in older age groups remains to be established. Notably, existing rVE estimates are restricted to head-to-head comparisons against sdIIVs, which are no longer the preferred standard of care for older adults in several national guidelines (16, 33, 35, 36). The magnitude of effect observed with the optimized mRNA-1010 in adults aged ≥50 years (125) and first-generation modRNA in younger adults (133) aligns with rVE trials comparing RIV4 with sdIIV4 (rVE 30.0% in adults aged ≥50 years) (94) and hdIIV3 with sdIIV3 (rVE 24.2% in adults aged ≥65 years) (53). Applying Bucher’s technique for indirect comparisons using sdIIV3 as a common comparator, Joshi et al. (165) leveraged individual-level data from the P304 study and a large US claims database to estimate the relative effectiveness of mRNA-1010 versus enhanced SIVs in preventing influenza-related healthcare encounters among adults aged ≥65 years during the 2024/2025 season. Following target trial emulation and inverse probability weighting procedures, the indirect relative effectiveness of mRNA-1010 versus enhanced SIVs was 12.82% (95% CI: –36.91%, 44.49%). While these findings offer valuable early insights, indirect comparisons cannot substitute for direct head-to-head evidence. Consequently, it remains unknown whether mRNA platforms offer incremental clinical benefit over established enhanced SIV formulations. In this context, the planned US real-world relative effectiveness study designed to evaluate mRNA-1010 against enhanced SIVs in thousands of older adults against medically attended, RT-PCR-confirmed influenza (126) will provide important comparative evidence to inform future policies.

Overall, mRNA seasonal influenza vaccines demonstrate an acceptable safety profile that aligns with approved COVID-19 mRNA platforms (118), whose broad safety profiles have been extensively established (15). Although mRNA vaccines display higher overall reactogenicity compared to conventional platforms, solicited ARs are predominantly transient and of mild-to-moderate severity, whereas vaccine-related SAEs remain very rare and comparable to rates observed with licensed SIV comparators across clinical trials (118, 119, 124, 125, 132, 133, 138, 140, 144, 146, 148, 149, 151). Nevertheless, this higher local and systemic reactogenicity may represent a practical barrier to widespread vaccine acceptance (164). In the general population, perceived or anticipated vaccine-associated AEs are strongly associated with reduced SIV uptake, with fear of side effects remaining one of the primary drivers of vaccine refusal (166, 167). This baseline barrier is further compounded by broader mRNA-specific hesitancy that emerged during the COVID-19 pandemic, driven by concerns regarding rapid development timelines, potential side effects, misinformation, and institutional or pharmaceutical distrust (168). Demonstrating persistent public caution toward novel platforms, a global survey revealed that 47.5% of respondents reported greater trust in traditional protein-based COVID-19 vaccines than in mRNA vaccines, while 39.0% were neutral and only 13.5% disagreed (169). Because direct recommendations from primary care physicians represent one of the most powerful enabling factors for influenza vaccination (170), general practitioners will play a crucial role in framing these risks. In this regard, provider-patient communication leveraging layperson concerns about the clinical disease burden of influenza and COVID-19, rather than focusing heavily on underlying platform technology, may be more effective in supporting vaccine uptake (169).

Vaccination coverage for both seasonal influenza and COVID-19 remains insufficient in most jurisdictions, presenting a persistent public health challenge. Across Europe during the 2024/2025 campaign, median SIV coverage among older adults reached only 47% (171), while COVID-19 booster uptake lagged at just 8.7% (172). Although SIV uptake temporarily increased during the initial phase of the COVID-19 pandemic, this trajectory was short-lived, with several countries experiencing declines as early as 2021 (173). Furthermore, despite public health guidelines authorizing the co-administration of SIV and COVID-19 vaccines during a single visit (174), the actual co-administration rates remain low (175, 176). In this context, combination mRNA vaccines, exemplified by the recently authorized mRNA-1083, have been developed to streamline annual respiratory prevention. In theory, a single-injection dual-target regimen could reduce appointment burdens, simplify cold-chain demands, and lower delivery costs while encouraging schedule adherence (145). Ecological data support a potential synergy, showing that COVID-19 vaccination uptake is consistently higher in populations with higher SIV coverage rates (177). A US survey highlighted potential behavioral synergy, concluding that a combination vaccine may slightly increase COVID-19 vaccination coverage: acceptance of a dual formulation (50%) surpassed willingness to receive a standalone COVID-19 booster (45%), despite remaining below a standalone SIV acceptance (58%) (178). Nevertheless, whether combination platforms will translate into meaningful population-level benefits remains unclear. Moreover, because authorization of mRNA-1083 relied on immunogenicity surrogates rather than efficacy endpoints (147), robust real-world post-marketing studies and health technology assessments are essential to evaluate its true impact on disease prevention, real-world compliance, and cost-effectiveness across distinct risk groups.

In summary, standalone and combination mRNA seasonal influenza platforms represent a significant technological leap by overcoming several drawbacks of the traditional egg-based platform and accelerating production timelines. While maintaining an acceptable safety profile, initial clinical data suggest these candidates offer advantages over sdIIVs in both immunogenicity and/or efficacy. Unlike the multidose vials used during the COVID-19 pandemic, leading mRNA vaccines are formulated in single-dose pre-filled syringes (130, 147), simplifying administration and streamlining logistics. Accumulating stability data have expanded refrigerated shelf life, rendering distribution increasingly compatible with standard cold-chain systems (112). However, translation to global health impact faces substantial socio-economic hurdles. Currently, enhanced SIVs (such as hdIIV, aIIV, and RIV) are deployed predominantly in high-income countries due to premium pricing structures. mRNA vaccines are poised to follow a similar trajectory, launching primarily in high-income markets at a higher cost than sdIIVs. To prevent widening the global divide in access to next-generation respiratory vaccines, sustainable technology transfer is critical (20).
