# Supplementary material for: mRNA-based seasonal influenza vaccines: an overview of immunogenicity, efficacy, and safety
Source: Front Immunol. 2026 Sep 14;17:1965512. doi: 10.3389/fimmu.2026.1965512 (PMC13617365; doi:10.3389/fimmu.2026.1965512)
Supplement: Supplementary file 1 [file Table1.docx]

Supplementary Material

**Supplementary Table 1.** Principal characteristics of trials on the efficacy, immunogenicity and safety of mRNA vaccines for seasonal influenza included in the review.

| **Study (Ref)** | **Phase** | **Design** | **Sponsor** | **Season (Hemisphere)** | **Population** | **mRNA vaccine** | **Licensed comparator** | **Primary objectives** |
| --- | --- | --- | --- | --- | --- | --- | --- | --- |
| 101, NCT04956575 (117, 118) | I/II | Randomized, controlled, observer-blind, dose-ranging | Moderna | Part 1: 2021 (Southern). Parts 2 and 3: 2021/2022 (Northern) | Healthy (part 1) or medically stable adults (parts 2 and 3) ≥18 years (*n* = 180 in part 1; *n* = 503 in part 2; *n* = 202 in part 3) | Part 1: original mRNA-1010 (50 *µ*g, 100 *µ*g, or 200 *µ*g). Part 2: original mRNA-1010 (25 *µ*g, 50 *µ*g, or 100 *µ*g). Part 3: original mRNA-1010 (6.25 *µ*g, 12.5 *µ*g, or 25 *µ*g). All vaccines were quadrivalent | Part 1: placebo. Parts 2 and 3: sdIIV4 | Safety, reactogenicity, immunogenicity |
| NCT05397223 (127–129) | I | Randomized, controlled, open-label | Moderna | 2022/2023 (Northern) | Healthy adults ≥18 years (*n* = 119)^1^ | Original mRNA-1010 (50 *µ*g, quadrivalent) | aIIV4 | Safety, reactogenicity |
| P301, NCT05415462 (119) | III | Randomized, controlled, observer-blind | Moderna | 2022 (Southern) | Adults ≥18 years (*n* = 6,102) | Original mRNA-1010 (50 *µ*g, quadrivalent) | sdIIV4 | Safety, reactogenicity, immunogenicity |
| P302, NCT05566639 (119) | III | Randomized, controlled, observer-blind | Moderna | 2022/2023 (Northern) | Adults ≥50 years (*n* = 22,502) | Original mRNA-1010 (50 *µ*g, quadrivalent) | sdIIV4 | Relative efficacy, safety, reactogenicity |
| Matz et al. (122) | NA | Observational | Moderna | 2022/2023 and 2023/2024 (Northern) | Healthy adults 20–50 years (*n* = 75) | mRNA-1010 (quadrivalent) | sdIIV4 | Immunogenicity (B cell response) |
| NCT05868382 (123) | II | Randomized, controlled, observer-blind, dose-ranging | Moderna | 2022/2023 (Northern) | Healthy adults 18–49 years (*n* = 270) | Original mRNA-1010 (50 *µ*g), optimized mRNA-1010 composition 1 (25 *µ*g or 50 *µ*g), optimized mRNA-1010 composition 2 (12.5 *µ*g, 25 *µ*g, or 50 *µ*g). All vaccines were quadrivalent | None | Safety, reactogenicity |
| P303, NCT05827978 (124) | III | Randomized, controlled, observer-blind | Moderna | Part A: 2022/2023 (Northern). Parts B and C: 2023/2024 (Northern) | Medically stable adults ≥18 years (part A, *n* = 2,414), 18–64 years (part B, *n* = 2,994), and ≥65 years (part C, *n* = 3,003) | Optimized mRNA-1010 (50 *µ*g, quadrivalent) | Parts A and B: sdIIV4. Part C: hdIIV4 | Part A: Safety, reactogenicity, immunogenicity. Parts B and C: Safety, reactogenicity, immunogenicity (non-inferiority) |
| P304 (Fluent), NCT06602024 (125, 126) | III | Randomized, controlled, double-blind | Moderna | 2024/2025 (Northern) | Medically stable adults ≥50 years (*n* = 40,805) | Optimized mRNA-1010 (37.5 *µ*g, trivalent) | sdIIV3 or sdIIV4 | Relative efficacy, safety, reactogenicity |
| C4781001, NCT05052697 (132) | I/II | Randomized, controlled, dose-ranging, single-blind | Pfizer | 2021/2022 (Northern) | Healthy or medically stable adults 65–85 years (substudy A, phase I; *n* = 436) and 18–85 years (substudy B, phase I/II; *n* = 614) | Substudy A: monovalent modRNA encoding influenza A (3.75 *µ*g, 7.5 *µ*g, 15 *µ*g, or 30 *µ*g); bivalent modRNA encoding influenza A (3.75 *µ*g, 7.5 *µ*g, or 15 *µ*g) and influenza B (7.5 *µ*g or 15 *µ*g); quadrivalent modRNA (30 *µ*g). Substudy B: modRNA (30 *µ*g or 60 *µ*g, quadrivalent) | hdIIV4 | Safety, reactogenicity |
| ISRCTN13789612 (135) | IIa | Randomized, controlled, double-blind, human challenge with an unblinded, unvaccinated external control group | Pfizer and hVIVO | 2022/2023 (Northern) | Healthy adults 18–55 years (*n* = 203) | Monovalent A(H1N1)pdm09 modRNA (7.5 *µ*g) | sdIIV4 | Absolute efficacy |
| C4781004, NCT05540522 (133, 134) | III | Randomized, controlled, observer-blind | Pfizer | 2022/2023 (Northern), 2023 (Southern) | Healthy or medically stable adults 18–64 (*n* = 18,476) years and ≥65 years (*n* = 27,291) | modRNA (30 *µ*g, quadrivalent)^2^ | sdIIV4 | Relative efficacy, safety, reactogenicity |
| NCT05446740 (138) | I | Randomized, controlled, dose-ranging, partially blinded^3^ | GSK and CureVac | 2021/2022 and 2022/2023 (Northern) | Healthy or medically stable adults 18–45 (*n* = 276) years and 60–80 years (*n* = 48) | Monovalent A(H1N1)pdm09 FLUmHA (younger adults: 0.5 *µ*g, 1 *µ*g, 2 *µ*g, 6 *µ*g, 9 *µ*g, 12 *µ*g, 18 *µ*g, 36 *µ*g, 54 *µ*g, or 100 *µ*g: older adults: 18 *µ*g) | sdIIV4 | Safety, reactogenicity, immunogenicity |
| NCT05827068 (140) | I/II | Randomized, controlled, open-label | Moderna | 2022/2023 (Northern) | Healthy adults 50–75 years (*n* = 699) | Original mRNA-1010 [50 *µ*g, quadrivalent, three formulations with alternative A(H3N2)]; mRNA-1011.1 [50 *µ*g, pentavalent with one additional A(H3N2)]; mRNA-1011.2 [40 *µ*g, quadrivalent with one additional A(H3N2) and no B/Yamagata]; mRNA-1012 [50 *µ*g or 62.5 *µ*g, pentavalent with two additional A(H3N2) and no B/Yamagata] | None | Safety, reactogenicity |
| NCT05333289 (144) | I/II | Randomized, controlled, observer-blind | Moderna | 2021/2022 (Northern) | Healthy adults 18–75 years (*n* = 572) | Original mRNA-1010 (50 *µ*g, quadrivalent); mRNA-1020 (50 *µ*g, 100 *µ*g, or 150 *µ*g, quadrivalent with HA:NA ratio 1:1); mRNA-1020 (25 *µ*g, 50 *µ*g, or 100 *µ*g, quadrivalent with HA:NA ratio ≈3:1) | RIV4 | Safety, reactogenicity |
| NCT05375838 (146) | I/II | Randomized, controlled, stratified, dose-ranging, observer-blind | Moderna | 2022 (Southern) | Healthy adults 18–75 years (*n* = 550) | First-generation mRNA-1273 COVID-19 vaccine (50 *µ*g); original mRNA-1010 (50 *µ*g); first-generation mRNA-1073 combination vaccine (25 *µ*g, 50 *µ*g, or 100 *µ*g). Seasonal influenza vaccine component of all vaccines was quadrivalent | None | Safety, reactogenicity |
| mRNA-1083-P101, NCT05827926 (148) | I/II | Randomized, controlled, dose-ranging, observer-blind | Moderna | 2022/2023 (Northern) | Healthy or medically stable adults 65–79 years (cohort A; *n* = 596) and 18–64 years (cohort B; *n* = 626) | Second-generation mRNA-1083 combination vaccines mRNA-1083.1 [15 *µ*g (cohort B only), 30 *µ*g (both cohorts), or 60 *µ*g (both cohorts); influenza HA:SARS-CoV-2 ratio 5:1], mRNA-1083.2 (27.5 *µ*g, 55 *µ*g, or 82 *µ*g; influenza HA:SARS-CoV-2 ratio ≈10:1], and mRNA-1083.3 (52.5 *µ*g; influenza HA:SARS-CoV-2 ratio 20:1]. Bivalent COVID-19 vaccines mRNA-1273 (50 *µ*g) and mRNA-1283 (10 *µ*g). Original (50 *µ*g) and optimized (50 *µ*g) mRNA-1010 influenza vaccines. Seasonal influenza vaccine component of all vaccines was quadrivalent | Cohort A: sdIIV4 and hdIIV4. Cohort B: sdIIV4 | Safety, reactogenicity |
| mRNA-1083-P301, NCT05827978 (149, 150) | III | Randomized, controlled, observer-blind | Moderna | 2023/2024 (Northern) | Adults ≥65 years (cohort A, *n* = 4,037) and 50–64 years (cohort B, *n* = 4,024) | Second-generation mRNA-1083 combination vaccine mRNA-1083 (40 *µ*g; four influenza strains); mRNA-1273 COVID-19 vaccine (50 *µ*g) co-administered with licensed influenza vaccine comparators | Cohort A: hdIIV4. Cohort B: sdIIV4 | Immunogenicity (non-inferiority), safety, reactogenicity |
| mRNA-1083-P302, NCT06694389 (151)^4^ | III | Randomized, controlled, observer-blind | Moderna | 2024/2025 (Northern) | Adults ≥50 years (*n* = 2,022) | Second-generation mRNA-1083 combination vaccine mRNA-1083 (40 *µ*g; four influenza strains); mRNA-1273 COVID-19 vaccine (50 *µ*g) co-administered with a licensed influenza vaccine comparator | sdIIV4 | Immunogenicity (non-inferiority), safety, reactogenicity |

^1^The study also included arms with non-influenza vaccines; the total number of subjects was 308.

^2^For older adults (≥65 years), the publicly posted ClinicalTrials.gov record identifies the modRNA arm as “Dose Level 2” and the comparator as a licensed quadrivalent influenza vaccine; the publicly displayed intervention record does not state the modRNA dose in micrograms or the comparator brand.

^3^The study was observer-blind with respect to the overall vaccine intervention (clinical evaluators and laboratory personnel were blinded, while vaccine-handling staff and the sponsor were not) and open-label regarding dose levels, with the lower-dose cohorts (0.5–12 *µ*g) conducted on a single-blind basis.

^4^Refers to part 1 of the trial conducted in Japan; results from parts 2 and 3 conducted in Taiwan and South Korea, respectively, were not reported in the publication.

HA, hemagglutinin; hdIIV4, high-dose quadrivalent egg-based inactivated split-virion influenza vaccine; NA, neuraminidase; RIV4, recombinant quadrivalent influenza vaccine; sdIIV3, standard-dose trivalent egg-based inactivated split-virion influenza vaccine; sdIIV4, standard-dose quadrivalent egg-based inactivated split-virion influenza vaccine.
